# VBP15, a Glucocorticoid Analogue, Is Effective at Reducing Allergic Lung Inflammation in Mice

**DOI:** 10.1371/journal.pone.0063871

**Published:** 2013-05-07

**Authors:** Jesse M. Damsker, Blythe C. Dillingham, Mary C. Rose, Molly A. Balsley, Christopher R. Heier, Alan M. Watson, Erik J. Stemmy, Roslyn A. Jurjus, Tony Huynh, Kathleen Tatem, Kitipong Uaesoontrachoon, Dana M. Berry, Angela S. Benton, Robert J. Freishtat, Eric P. Hoffman, John M. McCall, Heather Gordish-Dressman, Stephanie L. Constant, Erica K. M. Reeves, Kanneboyina Nagaraju

**Affiliations:** 1 ReveraGen BioPharma, Rockville, Maryland, United States of America; 2 Research Center for Genetic Medicine, Children's National Medical Center, Washington DC, United States of America; 3 Department of Integrative Systems Biology, Children's National Medical Center and George Washington University School of Medicine and Health Sciences, Washington DC, United States of America; 4 Department of Microbiology, Immunology, and Tropical Medicine, the George Washington University Medical Center, Washington DC, United States of America; 5 Endocrine Research Unit and the Australian National University Medical School, The Canberra Hospital, Australian Capital Territory, Australia; 6 PharMac LLC, Boca Grande, Florida, United States of America; University of Rochester Medical Center, United States of America

## Abstract

Asthma is a chronic inflammatory condition of the lower respiratory tract associated with airway hyperreactivity and mucus obstruction in which a majority of cases are due to an allergic response to environmental allergens. Glucocorticoids such as prednisone have been standard treatment for many inflammatory diseases for the past 60 years. However, despite their effectiveness, long-term treatment is often limited by adverse side effects believed to be caused by glucocorticoid receptor-mediated gene transcription. This has led to the pursuit of compounds that retain the anti-inflammatory properties yet lack the adverse side effects associated with traditional glucocorticoids. We have developed a novel series of steroidal analogues (VBP compounds) that have been previously shown to maintain anti-inflammatory properties such as NFκB-inhibition without inducing glucocorticoid receptor-mediated gene transcription. This study was undertaken to determine the effectiveness of the lead compound, VBP15, in a mouse model of allergic lung inflammation. We show that VBP15 is as effective as the traditional glucocorticoid, prednisolone, at reducing three major hallmarks of lung inflammation—NFκB activity, leukocyte degranulation, and pro-inflammatory cytokine release from human bronchial epithelial cells obtained from patients with asthma. Moreover, we found that VBP15 is capable of reducing inflammation of the lung *in vivo* to an extent similar to that of prednisone. We found that prednisolone–but not VBP15 shortens the tibia in mice upon a 5 week treatment regimen suggesting effective dissociation of side effects from efficacy. These findings suggest that VBP15 may represent a potent and safer alternative to traditional glucocorticoids in the treatment of asthma and other inflammatory diseases.

## Introduction

Asthma is a chronic multi-factorial disorder characterized by airway inflammation, hyper-responsiveness and mucus hypersecretion, which reflects remodeling of the airway epithelium and of the underlying basal lamina [1], [2]. Asthma is one of the most common airway inflammatory disorders and its incidence continues to increase in the United States [3]. The majority of asthma cases are mediated by an allergic response to environmental allergens, which results in degranulation of leukocytes such as eosinophils and mast cells [4], [5], [6], [7], [8], [9], bronchoconstriction, smooth muscle contraction, airway inflammation and mucus overproduction [10]. In addition, the asthmatic epithelium is inherently inflammogenic and upon injury (e.g. tobacco smoke, viral exposure, mechanical wounding) can release pro-inflammatory cytokines in the absence of inflammatory cells [11], [12], [13], [14], [15], [16].

Glucocorticoids are among the most prescribed drugs for therapeutic management of a wide variety of acute and chronic inflammatory conditions including lupus, myositis, rheumatoid arthritis, muscular dystrophy, and asthma [17], [18], [19], [20]. Despite their effectiveness, long-term treatment with glucocorticoids is limited by severe side effects including glaucoma, adrenal insufficiency, osteoporosis, cardiomyopathies, short stature, and mood or sleep disturbances [21], [22], [23], [24], [25]. In children with persistent asthma, inhaled glucocorticoids reduce growth during the first few years of therapy and height reduction persists throughout adulthood [26], [27].

The beneficial anti-inflammatory properties of glucocorticoids are due to multiple mechanisms [28]. Many occur shortly (<30 minutes) after glucocorticoid exposure. For example, glucocorticoids can inhibit inflammatory transcription factors such as NFκB and AP-1 via protein-protein signaling activity, resulting in decreased production of pro-inflammatory cytokines [29], [30], [31], [32]. Furthermore, glucocorticoids, acting via a non-genomic mechanism, can insert into cellular lipid bilayers and exert a biophysical effect on plasma membranes, affecting their structure and function, thus promoting membrane stability and even reducing cellular degranulation [33], [34], [35]. However, the most well-described mechanism of glucocorticoid action involves ligand binding to the cytoplasmic soluble glucocorticoid receptor (GR) to induce nuclear translocation of the ligand-receptor complex, which then interacts with glucocorticoid response elements (GREs) in the promoter regions of target genes, affecting transcription [36]. An increasing body of literature suggests that this transcriptional pathway is responsible for many of the adverse side effects associated with long-term glucocorticoid use [37], [38], [39].

Thus, a compound that maintains the beneficial anti-inflammatory properties of glucocorticoids but lacks the GRE-mediated transcriptional capabilities would represent an improved therapeutic option for patients suffering from inflammatory diseases. The potential for such a compound has not been well evaluated with regard to lung inflammation, although inhaled glucocorticoids contribute to systemic side-effects, and there is increasing concern about their safety for treatment of asthma in infants and young children [40], [41]. Therefore, we evaluated the effectiveness of VBP15 [42], the newly-identified lead compound selected from a previously described series of Δ9,11 glucocorticoid analogues [43], in a murine model of acute allergic lung inflammation and in differentiated human bronchial epithelial (HBE) cells obtained from patients diagnosed with asthma. Additionally, we assessed the potential of long-term VBP15 treatment to inhibit bone growth *in vivo* as a means of evaluating its ability to avoid detrimental glucocorticoid-related side-effects.

## Materials and Methods

### Ethics Statement

All animal work was conducted according to relevant national and international guidelines.

### Animals

For *in vivo* OVA-induced lung inflammation studies, female BALB/c mice at 6 weeks of age were purchased from Jackson Laboratories (Bar Harbor, Maine). For bone growth studies, timed-pregnant outbred CD-1 mice (e19) were purchased from Charles River Laboratories (Frederick, MD) and the male progeny were used for the experiment. All studies were reviewed and approved by the Institutional Animal Care and Use Committees at The George Washington University Medical Center and Children's National Medical Center.

### OVA-induced Model of Acute Allergic Lung Inflammation

The lung inflammation model used in the current studies has been previously described [44], [45]. Briefly, mice were primed via intraperitoneal (i.p.) injection with 50 µg of ovalbumin (OVA) in PBS with 100 µl of alum (200 µl total volume per mouse) on day 0. OVA-primed mice were challenged under light anesthesia (isoflurane) by intranasal delivery of 100 µg of OVA in PBS on days 7–10. Mice were sacrificed via CO_2_ exposure on day 12 for analysis. For *in vivo* intervention studies, mice (n = 5) received an oral dose of 20 mg/kg VBP-15 suspended in cherry syrup (30 µl total volume) on days 6–11. Additional groups of mice (n = 5) received 5 mg/kg of prednisone in cherry syrup as a positive control, or cherry syrup alone as a negative control. Following sacrifice on day 12, leukocytes were collected from the airways via bronchoalveolar lavage (BAL). For this procedure, a cannula was inserted into the trachea and two 1 ml washes of cold PBS were infused in and out of the airways. BAL fluid from individual mice was then centrifuged and supernatants were stored at −80°C for cytokine analysis. Following BAL, whole lungs were perfused via the right ventricle with 20 ml of cold PBS. For some experiments the lungs were then removed, chopped, and pushed through a metal strainer to generate single-cell suspensions. BAL and lung tissue cells were treated with ammonium chloride lysis buffer to remove red blood cells. The remaining leukocytes were then counted and stained for FACS analysis with a combination of PE-Cy5-anti-mouse CD4 and FITC-anti-mouse CD62L to identify effector/memory CD4^+^ cells (CD4^+^/CD62L^−^). Populations of eosinophils were identified using forward scatter/side scatter distribution, as previously described [44]. For studies addressing lung histopathology, 1 ml of 10% formalin was infused into the trachea following lung perfusion, and suture thread was used to tie off the inflated lungs. Fixed lungs were sent in 70% ethanol to Histoserv Inc. (Germantown, MD) for processing and staining with hematoxylin and eosin (H&E) and periodic acid Schiff (PAS). The frequency of PAS-positive airways was determined in a blinded manner by separately counting total airways and PAS-positive airways in each section via bright field microscopy.

### NFκB Inhibition Assay

The NFκB inhibition assay was modified from a previously described protocol [46]. Briefly, A549 epithelial cells (Panomics, Fremont, CA) stably transfected with a luciferase reporter construct regulated under NFκB response elements were grown in 75 mm^2^ flasks according to the manufacturer's instructions and transferred to 96-well plates upon reaching confluency. Once confluent, cells were serum starved for 48 hours and subsequently treated for 2 hours with increasing doses of VBP15, prednisolone (the active form of prednisone), or DMSO in serum-free medium at 37°C. Following treatment, cells were washed twice with PBS and exposed to TNF-α (100 ng/ml) for 6 hours in serum-free medium at 37°C. Cells were then washed once with PBS and lysed with lysis buffer (Promega Corp, Madison, WI) in order to measure luciferase activity with the Centro LB 960 luminometer (Berthold Technologies, GmbH & Co, Bad Wildbad, Germany).

### Human Bronchial Epithelial Cell Culture

Primary differentiated human bronchial epithelial (HBE) cells obtained from human asthma patients (n = 3) cultured in 12-well plates on collagen-coated Transwell membrane inserts at an air-liquid interface were obtained commercially (#AIR-606-Asthma; MatTek Corporation, Ashland, MA). Upon arrival, cells were washed in PBS, placed in proprietary medium provided by the manufacturer, and cultured for 16 hours at 37°C and 5% CO_2_ after which medium was replaced with identical medium lacking epidermal growth factor (EGF) and glucocorticoids. Cells were maintained under these conditions for an additional 22 hours before being pulse-treated at the basolateral surface for 2 hours with VBP-15 (10µM) or vehicle control (DMSO). Following pulsing, cells were placed in EGF/glucocorticoid-free medium, and 24 hours later, were pulse-treated again for 2 hours. Basolateral supernatant was removed and stored at −80°C for cytokine analysis.

### Measurement of Cytokines

BAL fluids from individual mice were concentrated 4-fold using 3-kDa cut-off Centricon columns (Millipore, Billerica, MA). Cytokines from BAL fluid and HBE supernatants were measured via cytometric bead assays (EBioscience, San Diego, CA) according to the manufacturer's protocol.

### RBL-2H3 Degranulation Assay

β-hexosaminidase release was used as an index of degranulation. Initially, RBL-2H3 cells (ATCC, Manassas, VA) were plated in 24-well tissue culture plates in growth media consisting of Dulbecco's Modified Eagle's Medium (DMEM), 10% FBS, and 1% penicillin-streptomycin. Cells were sensitized overnight with 1 µg/ml of anti-dinitrophenol (DNP) clone SPE-7 (Sigma-Aldrich, St. Louis, MO) at 37°C in growth media. Following sensitization, cells were washed with PBS, and Earl's Balanced Salt Solution (EBSS) (Invitrogen, Carlsbad, CA)+2.5% BSA was added to each well. Cells were then treated with VBP-15 (50 µM), prednisolone (50 µM), or an equal volume of DMSO for 7 minutes at 37°C. Subsequently, DNP-BSA (Sigma-Aldrich) at a concentration of 10 µg/ml was added to each well, and cells were incubated for an additional 20 minutes at 37°C. A well of untreated cells was lysed using 0.05% Triton-X-100 as a means of gauging total β-hexosamindase content. Supernatants and lysates were added to a 96-well plate (20 µl/well) and β-hexosaminidase substrate, p-nitrophenyl N-acetyl-beta-D-glucosamine (Sigma-Aldrich) in 0.05 M citrate buffer was added at a concentration of 1 mM in a volume of 20 µl to each well. EBSS+BSA and lysis buffer were also included on the plate as blank controls. The 96-well plate was incubated for 45 minutes at 37°C. Following incubation, 160 µl of sodium carbonate buffer (0.05 M) was added to each well to stop the reaction. The absorbance from each well was read at 405 nm using a microplate reader (Molecular Devices, Sunnyvale, CA). β-hexosaminidase release percentages were determined using the following formula: % = (Supernatant–blank)/(Total-blank) X 100. This assay was conducted in triplicate.

### Assessment of in vivo bone growth

Male outbred CD-1 mice (12 days of age) were treated orally with VBP15, prednisolone, or vehicle control (cherry syrup) daily for 5 weeks after which mice were sacrificed via CO_2_ and tibias were removed. Tibia lengths were measured via electronic caliper, with n = 10 mice/treatment group.

### Statistical Analysis

For most experiments, statistical significance was established using one-way ANOVA with post-hoc Tukey's test. For the NFκB inhibition experiment a one-way ANOVA with Bonferroni's post-hoc test was used. For HBE cell cytokine analysis, statistical significance was established by Student's T-test. All analyses were performed using Graphpad Prism software.

## Results

### VBP15 reduces lung inflammation in a murine model of allergic lung inflammation

**Figure 1:**
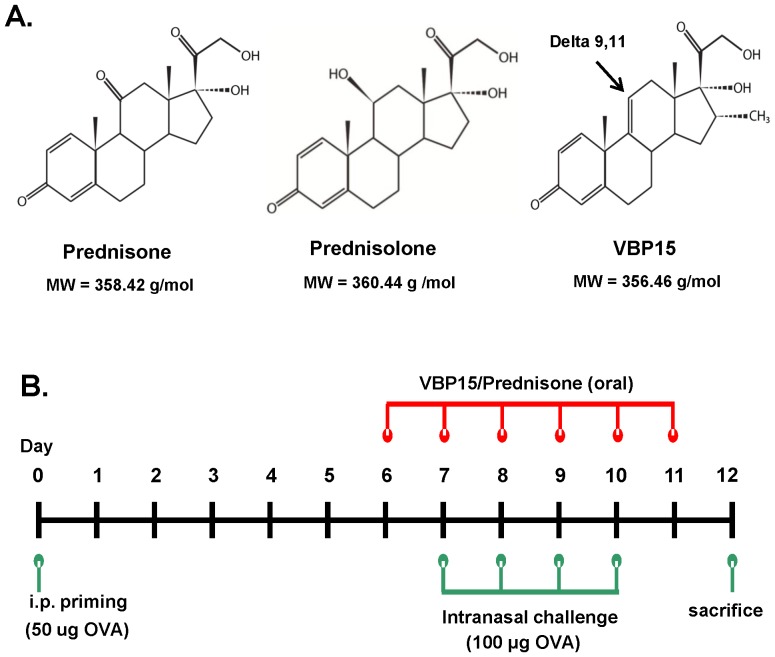
Structure of VBP15 and schematic of the OVA-induced model of acute allergic lung inflammation. (A) Chemical structures of prednisone (left panel), prednisolone (mid panel), and VBP15 (right panel). VBP compounds include a delta-9,11 double bond and tail group modifications. (B) Diagram of OVA-induced mouse model of allergic lung inflammation.

**Figure 2:**
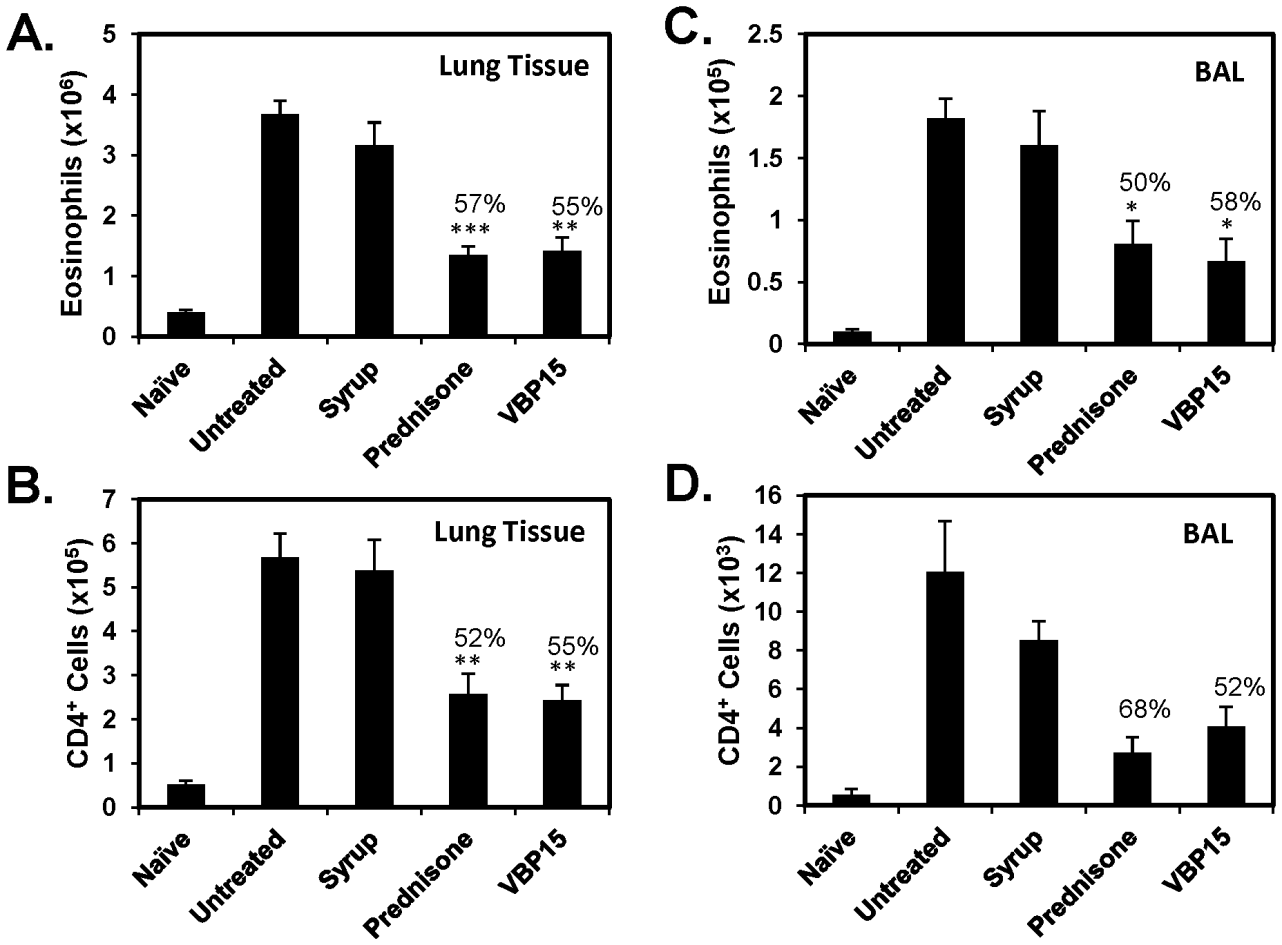
VBP15 reduces leukocyte infiltration in the OVA-induced model of acute allergic lung inflammation. OVA-challenged mice were either left untreated or treated with oral doses of prednisone, VBP15 (20 mg/kg), or cherry syrup alone daily for 6 days. A group of non-challenged mice (naïve) was included in order to assess basal inflammatory parameters. Lung tissue and BAL cells underwent FACS analysis to determine the number of infiltrating eosinophils (A and C) and effector/memory CD4+T cells (B and D). Bar graphs represent mean (±SE) cell numbers. Percentages indicate percentage reduction compared to vehicle control. *p<0.05; **p<0.01; ***p<.001 compared to syrup group with n = 5 mice per group.

**Figure 3:**
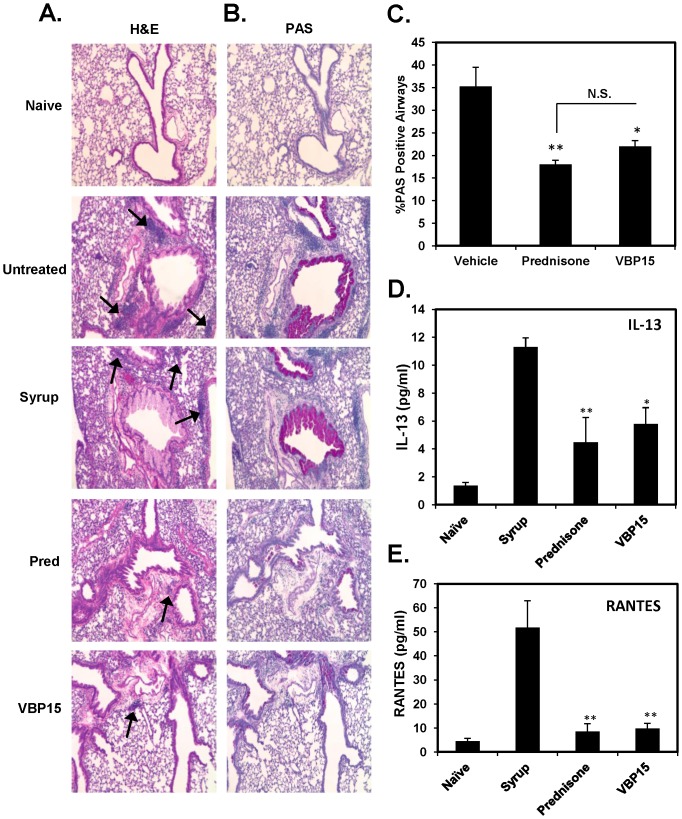
VBP15 reduces acute allergic lung inflammation. OVA-challenged mice were either left untreated or treated with oral doses of prednisone, VBP15 (20 mg/kg), or cherry syrup alone daily for 6 days. A group of non-challenged mice (naïve) was included in order to assess basal inflammatory parameters. Perfused whole lungs were processed for histological analysis and stained with H&E (A) or PAS (B). Images (10× magnification) represent areas of tissue surrounding bronchioles. Arrows on H&E sections indicate inflammatory foci. Percentage of PAS positive airways were counted via bright field microscopy (C). Bar graph represents mean (±SE)% PAS positive airways. *p<.05; **p<0.01 compared to the vehicle control group with n = 5 mice per group. IL-13 (D) and RANTES (E) were measured in BAL fluid by flow cytometric bead array. Bar graphs represent mean (±SE) cytokine concentration values. *p<.05; **p<0.01 compared to syrup group with n = 5 mice per group.

VBP compounds, in contrast to prednisone and prednisolone, contain a delta 9,11 bond (Figure 1A) that abolishes GRE-mediated gene transcription [43], [47]. In the current studies we wanted to determine if our lead compound, VBP15, would retain similar anti-inflammatory efficacy compared to prednisone in the widely used OVA-induced mouse model of allergic lung inflammation (Figure 1B). After a 6-day oral dosing regimen of VBP15, we observed a significant reduction similar to that of prednisone in the number of infiltrating eosinophils (55% reduction compared to vehicle control) (Figure 2A), as well as, effector/memory CD4+T cells (55% reduction) (Figure 2B) into the lung tissue of OVA-exposed and challenged mice. These changes in the lung were also reflected in the airways as there was a striking decrease in the presence of eosinophils (58% reduction) and CD4^+^ cells (52% reduction) in the BAL fluid (Figure 2C and D). Additionally, lung tissue sections stained with H&E to assess inflammatory pathology and PAS to assess for mucus overproduction/hypersecretion displayed a striking reduction in the presence of inflammatory foci and PAS-positive airways in VBP15-treated mice compared to mice treated with control cherry syrup alone (Figure 3A and 3B). VBP15 treatment induced a reduction in the percentage of PAS-positive airways that was comparable to that observed in tissue sections of prednisone-treated mice (Figure 3C). Furthermore, cytokine analysis of BAL fluid from both prednisone and VBP-treated mice demonstrated significant decreases in the inflammatory cytokine, IL-13 (50% reduction), and the T-cell chemokine, RANTES (81% reduction) (Figure 3D and 3E). These findings indicate that VBP15 is capable of reducing lung inflammation *in vivo* with a similar potency to that mediated by traditional glucocorticoids.

### VBP15 reduces NFκB activity

**Figure 4:**
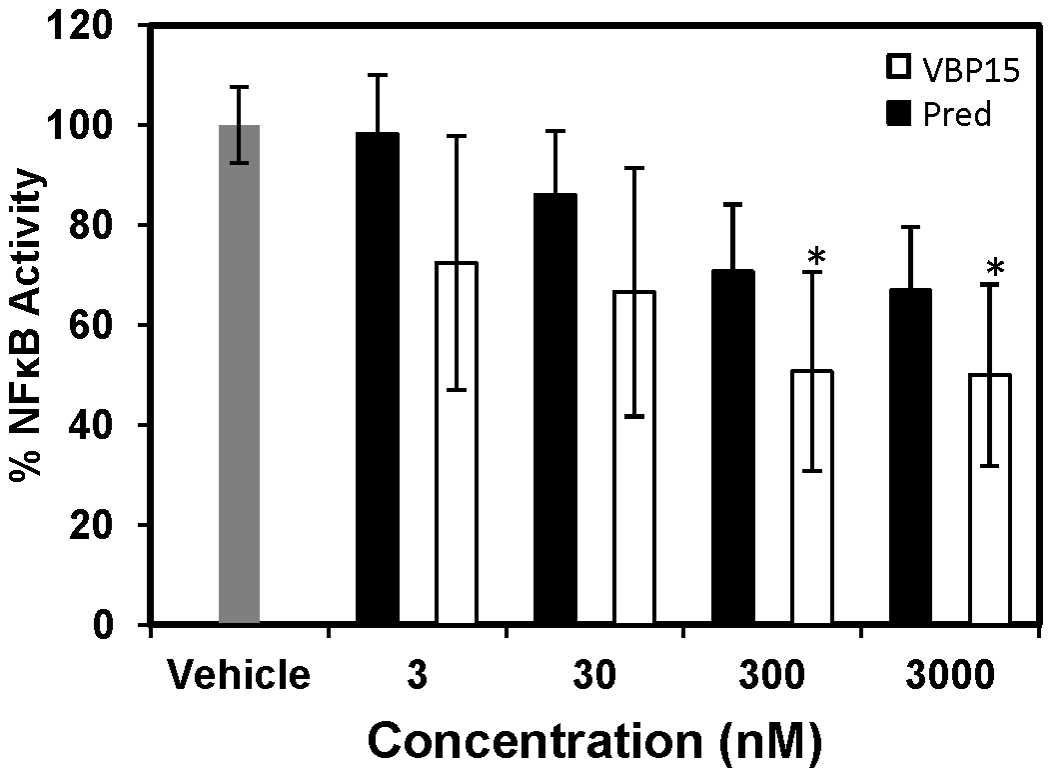
VBP15 inhibits NFκB activity. A549 cells stably-transfected with a luciferase NFκB construct were exposed to increasing concentrations of VBP15 or prednisolone (3, 30, 300, 3000 nM) followed by TNFα stimulation before measuring luciferase activity. Bar graph represents mean (±SE) luciferase units. *p<.012 (due to the Bonferroni adjustment for multiple comparisons) compared to treatment with vehicle control. Data represents 4 biological replicates with assay performed in triplicate.

Previous studies have shown that glucocorticoids can inhibit the activity of the pro-inflammatory transcription factor, NFκB, through a GRE-independent mechanism [47]. Thus, we determined whether VBP15 had the capacity to inhibit TNFα-induced NFκB expression in lung-epithelial cells to a similar extent as traditional glucocorticoids. To accomplish this, we made use of a TNF-α induced luciferase NFκB construct stably-transfected into A549 human lung epithelial cells. Interestingly, after pre-treating A549 cells with VBP15, we found that it had the capacity to reduce NFκB activity in a dose-response manner to a similar extent (56% reduction) to that observed with prednisolone, the active form of prednisone (Figure 4). Cell viability analysis via MTT assay revealed no significant differences between treatment groups (data not shown).

### VBP15 reduces leukocyte degranulation

**Figure 5:**
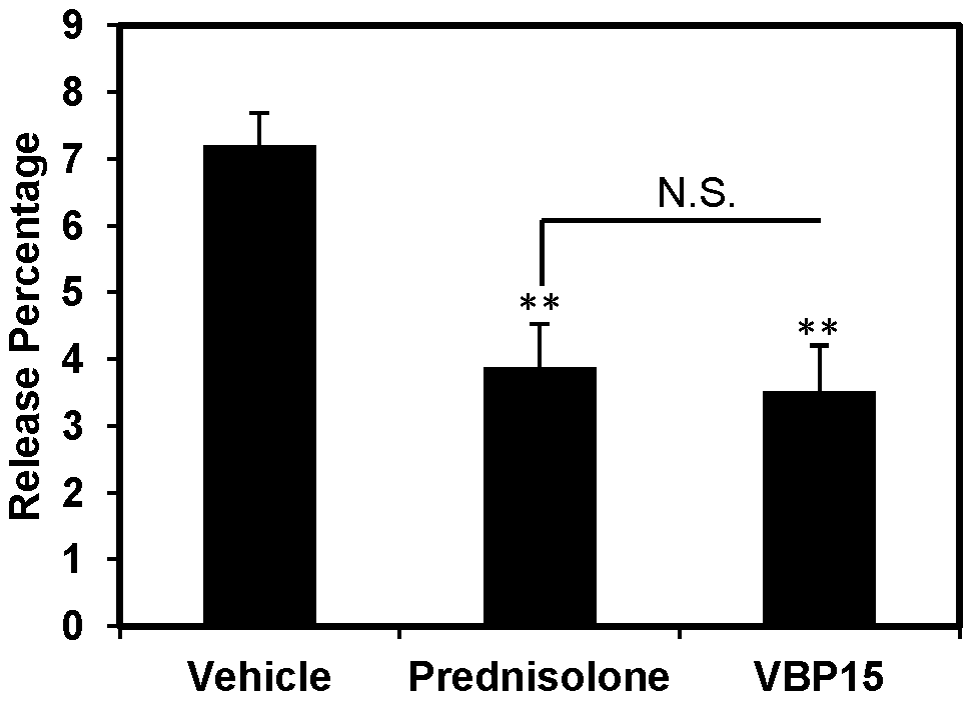
VBP15 reduces leukocyte degranulation. Anti-DNP-sensitized RBL-2H3 cells were treated with prednisolone (50 µM), VBP-15 (50 µM), or vehicle control (DMSO) for 7 minutes followed by addition of DNP to induce degranulation. The reaction was allowed to proceed for an additional 20 minutes before supernatant was removed and tested for β-hexosaminidase content. A well of untreated cells was lysed to gauge total β-hexosamindase content. Release percentage was determined using a formula described in Materials and Methods. Bar graph represents mean (±SE) release percentage. **p<0.01 compared to vehicle control. Data represents 3 biological replicates with assay performed in triplicate. N.S. = Not statistically significant.

Glucocorticoids have been shown to prevent degranulation of mast cells in a guinea pig model of allergic lung inflammation via a non-genomic mechanism possibly involving stabilization of the plasma membrane [34]. We wondered if VBP15 would likewise inhibit leukocyte degranulation. We addressed this question using the RBL-2H3 cell line, which has been extensively used for studying cellular degranulation. We observed that VBP15 significantly inhibited the release of β-hexosaminidase (51% reduction), a marker for cell degranulation, to an extent similar to that mediated by prednisolone (Figure 5). This finding supports the hypothesis that VBP15 is as effective as a traditional glucocorticoid at inhibiting cell degranulation, a well-described mechanism involved in the pathogenesis of allergic lung inflammation.

### VBP15 reduces inflammatory cytokine secretion from human epithelial cells

**Figure 6:**
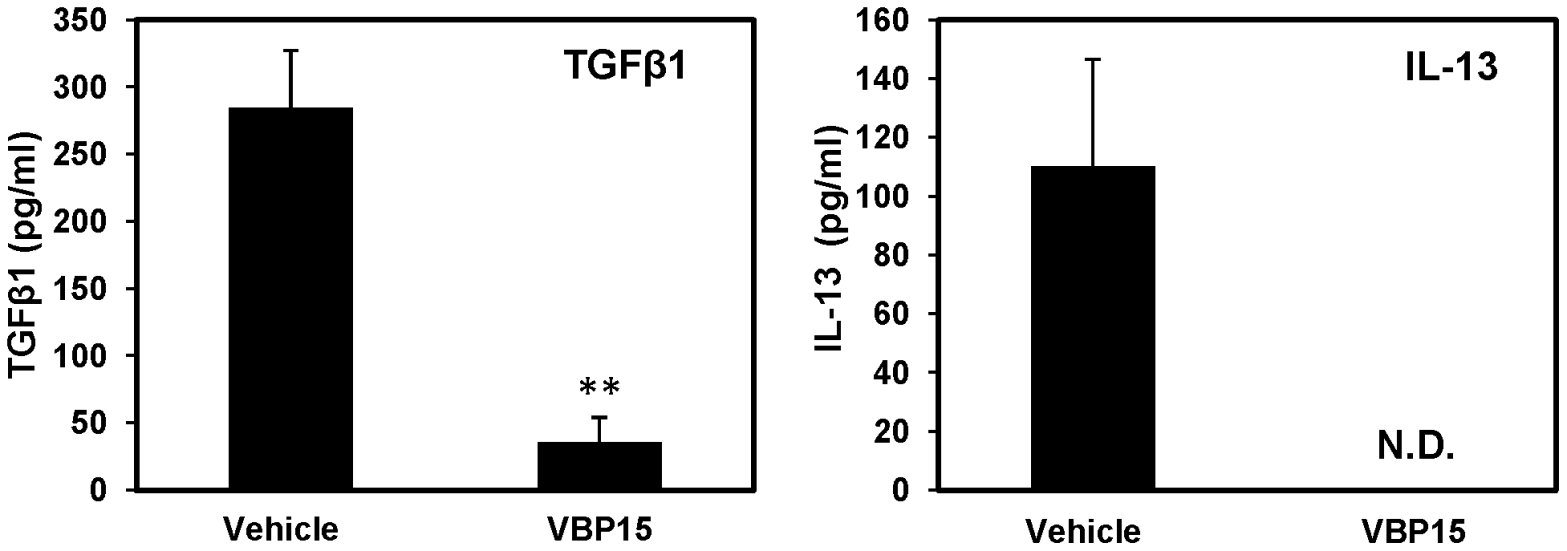
VBP15 reduces basolateral cytokine secretion from human bronchial epithelial cells obtained from asthmatic patients. HBE cells from 3 separate human donors were pulse-treated with VBP15 (10 µM) or vehicle control (DMSO). Basolateral surface supernatant was tested for the presence of TGFβ1 (left panel) and IL-13 (right panel) by flow cytometric bead array. Bar graphs represent mean (±SE) concentration values. **, *p*<0.01 compared to vehicle control with n = 3 donors. N.D. = Not Detectible (lower limit of detection = 4.5 pg/ml).

We next determined if VBP15 had an inhibitory effect on basolateral cytokine secretion in inflammatory epithelial cells from human bronchial epithelial cells (HBE) obtained from patients diagnosed with asthma, as we previously demonstrated with dexamethasone, a classical glucocorticoid [12]. After two short pulse treatments with VBP15, the basolateral secretion of TGFB1 and IL-13 from HBE cells was almost completely inhibited (87% and 100%, respectively) (Figure 6). Since epithelial cells isolated from asthma patients have been shown to release inflammatory cytokines in the absence of immune cells, this finding suggests that VBP15 is effective at targeting non-allergy-related mechanisms as well.

### VBP15 does not affect bone growth in vivo

**Figure 7:**
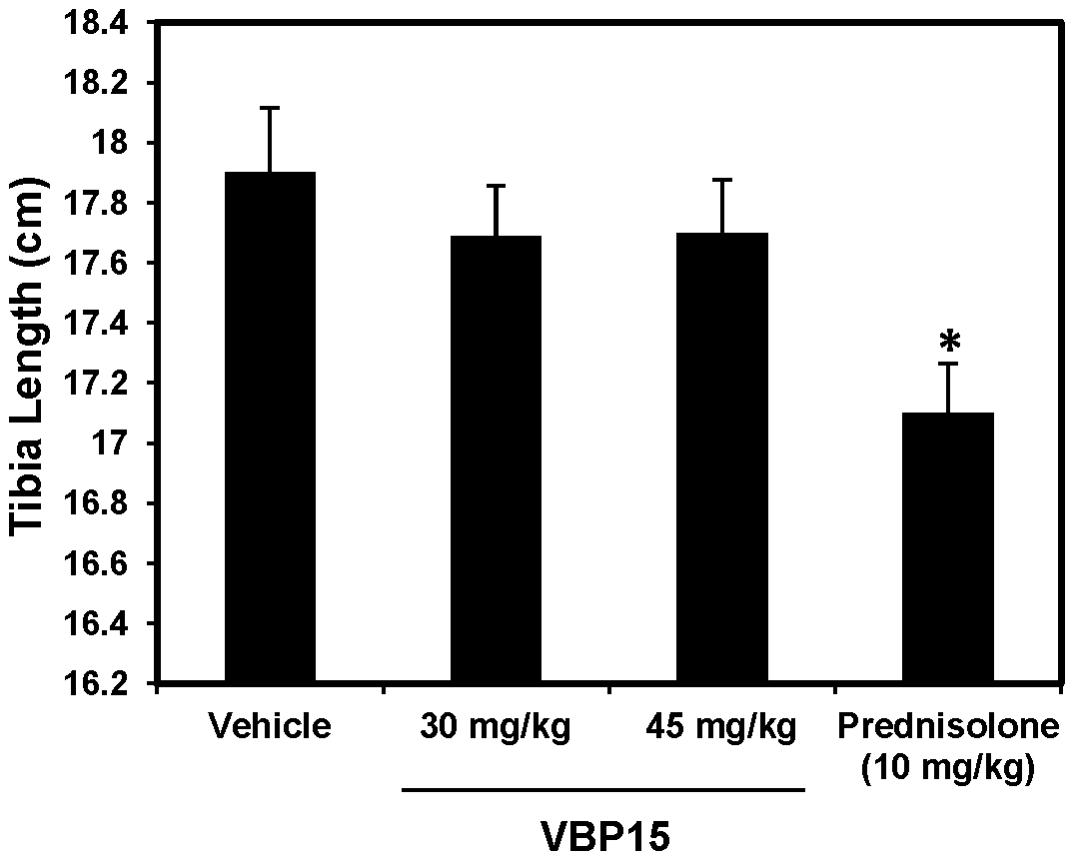
VBP15 does not induce tibia length shortening. Wildtype outbred CD1 mice were treated daily for 5 weeks with VBP15 (30 and 45 mg/kg), prednisolone (10 mg/kg) or vehicle control starting at 12 days of age. At the end of the treatment, tibias were harvested and measured. Bar graph represents mean (±SE) tibia length values. *p<0.05 compared to vehicle control with n = 10 mice/group.

One major known long term side-effect of glucocorticoids is the detrimental effect on bone growth. Thus, we determined whether or not extended VBP15 treatment could result in decreased bone growth by treating young mice (aged 12 days of age) daily for 5 weeks. We found that, contrary to mice treated with prednisolone, mice receiving VBP15 did not display significant tibia length shortening (Figure 7). Interestingly, we observed that doses of VBP15 four times higher than that of prednisolone did not result in any shortening of the tibia.

## Discussion

Asthma is a chronic inflammatory disease that affects over 300 million people worldwide in which the majority of cases are associated with an allergic response [48]. For the past 60 years, glucocorticoids have remained the standard of care for chronic, as well as acute asthma. Despite their effectiveness, severe side effects such as osteoporosis, cardiomyopathies, and glaucoma limit long-term use [21], [22], [23], [24], [25], [26], [27], [47], [49]. Since these detrimental effects are attributed to GRE-mediated gene transcription [50], we developed a series of steroidal analogues (VBP compounds) that lack the GRE-mediated transcriptional properties of traditional glucocorticoids but retain similar anti-inflammatory potential [43]. Therefore, we hypothesized that our lead VBP compound, VBP15 [42], when benchmarked against traditional glucocorticoids, would be just as effective in terms of its ability to reduce acute allergic lung inflammation *in vivo*.

Using an acute mouse model of allergic lung inflammation, we show that VBP15 is as effective as prednisone at reducing parameters of *in vivo* inflammation including leukocyte (eosinophils and CD4^+^ T cells) infiltration, tissue pathology, and mucus overproduction. *In vitro*, VBP15 was able to inhibit NFκB activity in lung epithelial cells to a similar degree to that seen with prednisolone (the active form of prednisone). Interestingly, we observed *in vivo* that both IL-13 and RANTES, two cytokines regulated by NFκB [51], [52] that also promote respective eosinophil and CD4^+^ T cell recruitment, were found to be reduced in the airways of VBP15 treated mice. Moreover, IL-13 is a known inducer of goblet cell metaplasia and mucus overproduction [53], [54] which was strikingly decreased in lungs of mice that were treated with VBP15. A well-documented anti-inflammatory effect of traditional glucocorticoids is that they inhibit the degranulation of leukocytes via non-genomic and non-GRE-related mechanisms [34]. We observed that VBP15 was capable of reducing leukocyte cell degranulation to a similar degree to that seen with prednisolone. Since RANTES and IL-13 are two products known to be released by degranulating leukocytes [55], [56] and are reduced in our *in vivo* studies, we believe that this is an additional mechanism whereby VBP15 exerts an anti-inflammatory effect within the context of acute allergic lung inflammation. It is important to note that conventional steroids may induce beneficial anti-inflammatory effects via their ability to cause GRE-mediated transcription. An example of this includes glucocorticoid-induced GRE-mediated production of IL-10 [57], [58], [59], a cytokine which has been shown to reduce allergic lung inflammation in mice [60]. However, despite lacking the ability to induce GRE-mediated transcription and potential IL-10 production, VBP15 still has the capacity to reduce allergic lung inflammation to a degree similar to that of conventional steroids. Mechanistic studies are currently underway addressing the effect of VBP15 on GR translocation and how it may affect a potential GR-NFκB protein interaction. Furthermore, we are planning on investigating how lung cells grown in hormone-depleted media respond to VBP15 treatment *in vitro*.

A major long term side-effect of glucocorticoids is their detrimental effect on bone growth, especially in children with asthma. Our data show that daily treatment of young mice with VBP15 at doses as high as 45 mg/kg does not inhibit bone growth of the tibia, in contrast to prednisolone. Doses of 20 mg/kg were shown to be anti-inflammatory in the OVA-induced mouse model of lung inflammation used in the current study, which mediates acute allergic responses. While similar acute responses are seen in human asthmatic patients during exacerbations, the human disease is predominantly chronic. Indeed, the acute *in vivo* model used in this study has some limitations. For example, in contrast to the human disease, airway remodeling changes accompanied by collagen deposition and fibrosis are not observed in this model. However, these changes are observed in a chronic model of allergic lung inflammation [61]. Studies to establish the potential efficacy of VBP compounds using this chronic model are currently underway. These studies will also enable us to assess *in vivo* the absence or presence of additional negative side effects traditionally seen with long-term use of glucocorticoids in chronic asthma.

The pathogenesis of asthma is complex and heterogeneous. While allergy and the immune response are known to trigger a majority of asthma cases [5], [6], [9], other mechanisms of pathogenesis have been described. For example, a recent study demonstrates that HBE cells of asthmatic patients possess intrinsic inflammatory properties and have the capacity to release pro-inflammatory cytokines including IL-13 and TGFβ1 from their basolateral surface without any contribution from immune cells. Interestingly, exposure of HBE cells to pulse-treatment of traditional glucocorticoids was able to significantly reduce basolateral secretion of both IL-13 and TGFβ1 [12]. We demonstrate in the current study that VBP compounds were able to almost completely eliminate basolateral secretion of IL-13 and TGFβ1 from HBE cells that were obtained from asthma patients, suggesting that these compounds may inhibit pathogenic mechanisms in addition to those associated with allergy. Ongoing work is currently in progress aimed at further characterizing the function of these cells in response to VBP15 treatment. A bulk of the non-steroidal treatment options in the clinic revolves around targeting the allergic response as a means of treating asthma (e.g. mast cell stabilizers)[62], [63]. Since VBP15 may treat multiple mechanisms of asthma, and since they potentially lack the adverse glucocorticoid side effects, we believe that this compound may represent an efficacious and safer alternative to traditional glucocorticoids in the treatment of asthma and other inflammatory diseases.

## References

[1] Busse W, Banks-Schlegel S, Noel P, Ortega H, Taggart V, et al (2004) Future research directions in asthma: an NHLBI Working Group report. Am J Respir Crit Care Med 170: 683–690. 15215155 10.1164/rccm.200311-1539WS

[2] Busse W, Elias J, Sheppard D, Banks-Schlegel S (1999) Airway remodeling and repair. Am J Respir Crit Care Med 160: 1035–1042. 10471638 10.1164/ajrccm.160.3.9902064

[3] Akinbami LJ, Moorman JE, Liu X (2011) Asthma prevalence, health care use, and mortality: United States, 2005–2009. Natl Health Stat Report: 1–14. 21355352

[4] Larche M, Robinson DS, Kay AB (2003) The role of T lymphocytes in the pathogenesis of asthma. J Allergy Clin Immunol 111: 450–463; quiz 464. 12642820 10.1067/mai.2003.169

[5] Cohn L, Elias JA, Chupp GL (2004) Asthma: mechanisms of disease persistence and progression. Annu Rev Immunol 22: 789–815. 15032597 10.1146/annurev.immunol.22.012703.104716

[6] Mayr SI, Zuberi RI, Zhang M, de Sousa-Hitzler J, Ngo K, et al (2002) IgE-dependent mast cell activation potentiates airway responses in murine asthma models. J Immunol 169: 2061–2068. 12165533 10.4049/jimmunol.169.4.2061

[7] Holgate ST, Robinson C, Church MK (1988) The contribution of mast cell mediators to acute allergic reactions in human skin and airways. Allergy 43 Suppl 522–31. 2451448 10.1111/j.1398-9995.1988.tb05044.x

[8] Galli SJ, Costa JJ (1995) Mast-cell-leukocyte cytokine cascades in allergic inflammation. Allergy 50: 851–862. 8748716 10.1111/j.1398-9995.1995.tb02490.x

[9] Robinson DS, Hamid Q, Ying S, Tsicopoulos A, Barkans J, et al (1992) Predominant TH2-like bronchoalveolar T-lymphocyte population in atopic asthma. N Engl J Med 326: 298–304. 1530827 10.1056/NEJM199201303260504

[10] Wills-Karp M (1999) Immunologic basis of antigen-induced airway hyperresponsiveness. Annu Rev Immunol 17: 255–281. 10358759 10.1146/annurev.immunol.17.1.255

[11] Freishtat RJ, Nagaraju K, Jusko W, Hoffman EP (2010) Glucocorticoid efficacy in asthma: is improved tissue remodeling upstream of anti-inflammation. J Investig Med 58: 19–22. 10.231/JIM.0b013e3181b91654 PMC3324850 19730133

[12] Freishtat RJ, Watson AM, Benton AS, Iqbal SF, Pillai DK, et al.. (2011) Asthmatic Airway Epithelium is Intrinsically Inflammatory and Mitotically Dyssynchronous. Am J Respir Cell Mol Biol. 10.1165/rcmb.2010-0029OC PMC3135846 20705942

[13] Kicic A, Sutanto EN, Stevens PT, Knight DA, Stick SM (2006) Intrinsic biochemical and functional differences in bronchial epithelial cells of children with asthma. Am J Respir Crit Care Med 174: 1110–1118. 16908868 10.1164/rccm.200603-392OC

[14] Holgate ST, Lackie PM, Davies DE, Roche WR, Walls AF (1999) The bronchial epithelium as a key regulator of airway inflammation and remodelling in asthma. Clin Exp Allergy 29 Suppl 290–95. 10421830 10.1046/j.1365-2222.1999.00016.x

[15] Davies DE, Holgate ST (2002) Asthma: the importance of epithelial mesenchymal communication in pathogenesis. Inflammation and the airway epithelium in asthma. Int J Biochem Cell Biol 34: 1520–1526. 12379273 10.1016/s1357-2725(02)00048-1

[16] Hackett TL, Singhera GK, Shaheen F, Hayden P, Jackson GR, et al (2011) Intrinsic phenotypic differences of asthmatic epithelium and its inflammatory responses to respiratory syncytial virus and air pollution. Am J Respir Cell Mol Biol 45: 1090–1100. 21642587 10.1165/rcmb.2011-0031OC

[17] Tuckermann J, Bourguet W, Mandrup S (2010) Meeting report: nuclear receptors: transcription factors and drug targets connecting basic research with translational medicine. Mol Endocrinol 24: 1311–1321. 20519330 10.1210/me.2010-0083 PMC5417462

[18] Hillier SG (2007) Diamonds are forever: the cortisone legacy. J Endocrinol 195: 1–6. 17911391 10.1677/JOE-07-0309

[19] Larj MJ, Bleecker ER (2004) Therapeutic responses in asthma and COPD. Corticosteroids Chest 126: 138S–149S; discussion 159S–161S. 10.1378/chest.126.2_suppl_1.138S 15302774

[20] Expert Panel Report 3 (EPR-3): Guidelines for the Diagnosis and Management of Asthma-Summary Report 2007. J Allergy Clin Immunol 120: S94–138. 17983880 10.1016/j.jaci.2007.09.043

[21] Garbe E, LeLorier J, Boivin JF, Suissa S (1997) Inhaled and nasal glucocorticoids and the risks of ocular hypertension or open-angle glaucoma. JAMA 277: 722–727. 9042844

[22] Wordinger RJ, Clark AF (1999) Effects of glucocorticoids on the trabecular meshwork: towards a better understanding of glaucoma. Prog Retin Eye Res 18: 629–667. 10438153 10.1016/s1350-9462(98)00035-4

[23] Manzur AY, Kuntzer T, Pike M, Swan A (2008) Glucocorticoid corticosteroids for Duchenne muscular dystrophy. Cochrane Database Syst Rev: CD003725. 10.1002/14651858.CD003725.pub3 18254031

[24] Rehman Q, Lane NE (2003) Effect of glucocorticoids on bone density. Med Pediatr Oncol 41: 212–216. 12868121 10.1002/mpo.10339

[25] Ito T, Murata M, Kamiyama A (1979) Experimental study of cardiomyopathy induced by glucocorticoids. Jpn Circ J 43: 1043–1047. 522233 10.1253/jcj.43.1043

[26] Sharek PJ, Bergman DA (2000) The effect of inhaled steroids on the linear growth of children with asthma: a meta-analysis. Pediatrics 106: E8. 10878177 10.1542/peds.106.1.e8

[27] Kelly HW, Sternberg AL, Lescher R, Fuhlbrigge AL, Williams P, et al (2012) Effect of inhaled glucocorticoids in childhood on adult height. N Engl J Med 367: 904–912. 22938716 10.1056/NEJMoa1203229 PMC3517799

[28] Rhen T, Cidlowski JA (2005) Antiinflammatory action of glucocorticoids-new mechanisms for old drugs. N Engl J Med 353: 1711–1723. 16236742 10.1056/NEJMra050541

[29] Herrlich P (2001) Cross-talk between glucocorticoid receptor and AP-1. Oncogene 20: 2465–2475. 11402341 10.1038/sj.onc.1204388

[30] Coghlan MJ, Elmore SW, Kym PR, Kort ME (2003) The pursuit of differentiated ligands for the glucocorticoid receptor. Curr Top Med Chem 3: 1617–1635. 14683518 10.2174/1568026033451718

[31] Baudy AR, Saxena N, Gordish H, Hoffman EP, Nagaraju K (2009) A robust in vitro screening assay to identify NF-kappaB inhibitors for inflammatory muscle diseases. Int Immunopharmacol 9: 1209–1214. 19596085 10.1016/j.intimp.2009.07.001 PMC2745946

[32] van der Burg B, Liden J, Okret S, Delaunay F, Wissink S, et al (1997) Nuclear factor-kappa B repression in antiinflammation and immunosuppression by glucocorticoids. Trends Endocrinol Metab 8: 152–157. 18406801 10.1016/s1043-2760(97)00006-4

[33] Epps DE, McCall JM (1997) Physical and Chemical Mechanisms of the Antioxidant Action of Tirilazad Mesylate. In: Packer L, Cadenas E, editors. Handbook of Novel Antioxidants. New York: Marcel Dekker. pp. 95–137.

[34] Zhou J, Liu DF, Liu C, Kang ZM, Shen XH, et al (2008) Glucocorticoids inhibit degranulation of mast cells in allergic asthma via nongenomic mechanism. Allergy 63: 1177–1185. 18699934 10.1111/j.1398-9995.2008.01725.x

[35] Liu L, Wang YX, Zhou J, Long F, Sun HW, et al (2005) Rapid non-genomic inhibitory effects of glucocorticoids on human neutrophil degranulation. Inflamm Res 54: 37–41. 15723203 10.1007/s00011-004-1320-y

[36] Truss M, Beato M (1993) Steroid hormone receptors: interaction with deoxyribonucleic acid and transcription factors. Endocr Rev 14: 459–479. 8223341 10.1210/edrv-14-4-459

[37] Reichardt HM, Kaestner KH, Tuckermann J, Kretz O, Wessely O, et al (1998) DNA binding of the glucocorticoid receptor is not essential for survival. Cell 93: 531–541. 9604929 10.1016/s0092-8674(00)81183-6

[38] Reichardt HM, Tuckermann JP, Gottlicher M, Vujic M, Weih F, et al (2001) Repression of inflammatory responses in the absence of DNA binding by the glucocorticoid receptor. EMBO J 20: 7168–7173. 11742993 10.1093/emboj/20.24.7168 PMC125338

[39] Newton R, Holden NS (2007) Separating transrepression and transactivation: a distressing divorce for the glucocorticoid receptor? Mol Pharmacol 72: 799–809. 17622575 10.1124/mol.107.038794

[40] Adcock IM, Caramori G, Chung KF (2008) New targets for drug development in asthma. Lancet 372: 1073–1087. 18805336 10.1016/S0140-6736(08)61449-X

[41] Castro-Rodriguez JA, Pedersen S (2013) The role of inhaled corticosteroids in management of asthma in infants and preschoolers. Curr Opin Pulm Med 19: 54–59. 23143197 10.1097/MCP.0b013e32835b1165

[42] Reeves EK, Hoffman EP, Nagaraju K, Damsker JM, McCall JM (2013) VBP15: Preclinical characterization of a novel anti-inflammatory delta 9,11 steroid. Bioorg Med Chem 21: 2241–2249. 23498916 10.1016/j.bmc.2013.02.009 PMC4088988

[43] Baudy AR, Reeves EK, Damsker JM, Heier C, Garvin LM, et al (2012) Delta-9,11 modification of glucocorticoids dissociates nuclear factor-kappaB inhibitory efficacy from glucocorticoid response element-associated side effects. J Pharmacol Exp Ther 343: 225–232. 22743576 10.1124/jpet.112.194340 PMC3464029

[44] Balsley MA, Malesevic M, Stemmy EJ, Gigley J, Jurjus RA, et al (2010) A cell-impermeable cyclosporine A derivative reduces pathology in a mouse model of allergic lung inflammation. J Immunol 185: 7663–7670. 21057089 10.4049/jimmunol.1001707 PMC3603141

[45] Gwinn WM, Damsker JM, Falahati R, Okwumabua I, Kelly-Welch A, et al (2006) Novel approach to inhibit asthma-mediated lung inflammation using anti-CD147 intervention. J Immunol 177: 4870–4879. 16982929 10.4049/jimmunol.177.7.4870 PMC2855298

[46] King EM, Holden NS, Gong W, Rider CF, Newton R (2009) Inhibition of NF-kappaB-dependent transcription by MKP-1: transcriptional repression by glucocorticoids occurring via p38 MAPK. J Biol Chem 284: 26803–26815. 19648110 10.1074/jbc.M109.028381 PMC2785369

[47] De Bosscher K (2009) Selective Glucocorticoid Receptor modulators. J Steroid Biochem Mol Biol 120: 96–104. 10.1016/j.jsbmb.2010.02.027 20206690

[48] Dougherty RH, Fahy JV (2009) Acute exacerbations of asthma: epidemiology, biology and the exacerbation-prone phenotype. Clin Exp Allergy 39: 193–202. 19187331 10.1111/j.1365-2222.2008.03157.x PMC2730743

[49] Chrousos GA, Kattah JC, Beck RW, Cleary PA (1993) Side effects of glucocorticoid treatment. Experience of the Optic Neuritis Treatment Trial. JAMA 269: 2110–2112. 8468765

[50] Stahn C, Lowenberg M, Hommes DW, Buttgereit F (2007) Molecular mechanisms of glucocorticoid action and selective glucocorticoid receptor agonists. Mol Cell Endocrinol 275: 71–78. 17630118 10.1016/j.mce.2007.05.019

[51] Hinz M, Lemke P, Anagnostopoulos I, Hacker C, Krappmann D, et al (2002) Nuclear factor kappaB-dependent gene expression profiling of Hodgkin's disease tumor cells, pathogenetic significance, and link to constitutive signal transducer and activator of transcription 5a activity. J Exp Med 196: 605–617. 12208876 10.1084/jem.20020062 PMC2194004

[52] Moriuchi H, Moriuchi M, Fauci AS (1997) Nuclear factor-kappa B potently up-regulates the promoter activity of RANTES, a chemokine that blocks HIV infection. J Immunol 158: 3483–3491. 9120310

[53] Wills-Karp M, Luyimbazi J, Xu X, Schofield B, Neben TY, et al (1998) Interleukin-13: central mediator of allergic asthma. Science 282: 2258–2261. 9856949 10.1126/science.282.5397.2258

[54] Grunig G, Warnock M, Wakil AE, Venkayya R, Brombacher F, et al (1998) Requirement for IL-13 independently of IL-4 in experimental asthma. Science 282: 2261–2263. 9856950 10.1126/science.282.5397.2261 PMC3897229

[55] Ying S, Meng Q, Taborda-Barata L, Corrigan CJ, Barkans J, et al (1996) Human eosinophils express messenger RNA encoding RANTES and store and release biologically active RANTES protein. Eur J Immunol 26: 70–76. 8566086 10.1002/eji.1830260111

[56] Gessner A, Mohrs K, Mohrs M (2005) Mast cells, basophils, and eosinophils acquire constitutive IL-4 and IL-13 transcripts during lineage differentiation that are sufficient for rapid cytokine production. J Immunol 174: 1063–1072. 15634931 10.4049/jimmunol.174.2.1063

[57] Kube D, Platzer C, von Knethen A, Straub H, Bohlen H, et al (1995) Isolation of the human interleukin 10 promoter. Characterization of the promoter activity in Burkitt's lymphoma cell lines. Cytokine 7: 1–7. 7749063 10.1006/cyto.1995.1001

[58] Eskdale J, Kube D, Tesch H, Gallagher G (1997) Mapping of the human IL10 gene and further characterization of the 5′ flanking sequence. Immunogenetics 46: 120–128. 9162098 10.1007/s002510050250

[59] John M, Lim S, Seybold J, Jose P, Robichaud A, et al (1998) Inhaled corticosteroids increase interleukin-10 but reduce macrophage inflammatory protein-1alpha, granulocyte-macrophage colony-stimulating factor, and interferon-gamma release from alveolar macrophages in asthma. Am J Respir Crit Care Med 157: 256–262. 9445307 10.1164/ajrccm.157.1.9703079

[60] Campbell JD, Buckland KF, McMillan SJ, Kearley J, Oldfield WL, et al (2009) Peptide immunotherapy in allergic asthma generates IL-10-dependent immunological tolerance associated with linked epitope suppression. J Exp Med 206: 1535–1547. 19528258 10.1084/jem.20082901 PMC2715096

[61] McMillan SJ, Lloyd CM (2004) Prolonged allergen challenge in mice leads to persistent airway remodelling. Clin Exp Allergy 34: 497–507. 15005746 10.1111/j.1365-2222.2004.01895.x PMC3428844

[62] Kabra SK, Lodha R (2003) Long-term management of asthma. Indian J Pediatr 70: 63–72. 12619955 10.1007/BF02722747

[63] Renzi PM (1999) Antileukotriene agents in asthma: the dart that kills the elephant? CMAJ 160: 217–223. 9951445 PMC1229994

